# Optimization of absorption coefficient of quantum dot structures for infrared spectroscopy

**DOI:** 10.1038/s41598-025-19607-1

**Published:** 2025-09-18

**Authors:** Sameh A. Dakroury, Mohamed I. Wafa, Yasser M. El-Batawy, Nouran M. Ali

**Affiliations:** 1 Department of Engineering Math and Physics, Faculty of Engineering, Cairo University, Giza, 12613 Egypt; 2 Nanoelectronics Integrated Systems Center, Nile University, Giza, 12588 Egypt

**Keywords:** Self-assembled QD, IR photodetection, Nelder–Mead simplex algorithm, Bound to bound, QDIP, Sensitivity, Chemistry, Materials science, Optics and photonics, Physics

## Abstract

Infrared spectroscopy is a powerful tool used in chemical analysis and identification, material and polymer characteristics, pharmaceuticals and medical diagnostics, food industry, and environmental applications. Quantum Dots have shown significant potential as a top candidate for infrared photodetection of the transmitted and absorbed frequencies which is one of the main processes in IR spectroscopy. Therefore, the demand for accurate optimization techniques for enhanced detection is critically needed. In this work, we have developed an optimization study of the optical absorption coefficient of InAs/GaAs self-assembled quantum dots for IR photodetection specially in fingerprint region, where the Bound-to-bound absorption coefficient calculations are based on the bounded states estimation using the effective mass Hamiltonian diagonalization. Then, optimization has been performed which is based on the Nelder–Mead simplex algorithm where the objective function is maximizing the optical absorption coefficient at certain wavenumbers of interest of 600 and 800 cm^−1^. Also, the optimized absorption has been compared with previously published results for different dot shapes; semi-spherical, conical and truncated conical dots, showing a considerable enhancement of the optical absorption coefficient at the wavelengths of interest. A 5% sensitivity analysis has been performed for each QD cell parameters to study the effects of tolerances around the optimized design parameters. The presented optimization approach is generic that can be applied for different wavelengths, different QD structures, and different QD and barrier materials.

## Introduction

IR spectra provide valuable information about molecular vibrations, and these characteristic bands help identify functional groups or detect impurities. IR spectroscopy has several applications including phototoxicity^1^, DNA detecting^2,3^, biomedical^4,5^ and food quality control^6^, chemical analysis and identification, material and polymer characteristics^7^, pharmaceuticals and medical diagnostics^8^, food industry^9^, and environmental applications^10^. Generally, IR spectroscopy is based on applying IR radiation over the sample of interest and photodetection of the non-absorbed infrared radiation. When a chemical sample is exposed to IR radiation, it can absorb or reflect some frequencies and transmit the rest. The detector detects the transmitted frequencies and consequently reveals the values of the absorbed frequencies. In clinical spectroscopy, where the IR and Raman can be used to analyze and characterize tissues, cells, blood, urine, and saliva where these analyses and characteristic spectra indicate the contents of biomolecules such as proteins, carbohydrates, lipids and nucleic acids. Therefore, this spectroscopy can be used effectively for early detection, diagnosis or monitoring of human diseases such as Alzheimer, kidney and heart diseases^11–13^. Also, it can be used for characteristics of the fingerprint region, which is typically found between $\:500\:\text{cm}^{-1}\:$and$\:\:1500\:\text{cm}^{-1}$ on the IR spectrum^14^. Although, it is significant for providing a unique pattern for each individual molecule and identifying specific molecules in a compound or detecting impurities, the region is complex and crowded with peaks. The complexity of the fingerprint region is challenging because of the number of peaks which are generally due to carbon-carbon and carbon-hydrogen bond vibrations. This complexity gives it its unique identifying power and makes it a key tool in chemistry^15^.

Quantum Dots (QDs) are zero-dimensional nanostructures which confine charges in all directions with advantages of bandgap tunability, low-cost synthesis, and good tunable absorption and emission wavelengths^16–18^. III-V dots has been presented as promising alternatives to lead/cadmium due to the lower toxicity and tunable optoelectronic properties, which make it more suitable for photodetection applications such as light emitting devices and near infrared imaging as stated in the recent reviews^19,20^. Focusing on InAs/GaAs as one of the most common fabricated materials for self-assembled quantum dots^21–25^ which has been acknowledged as a high potential candidate for IR photodetection. Also, other III-V compounds were studied for photodetection such as InSb/InP^26^, GaSb/GaAs^27^, ZnTe^28^, InSb/GaSb, and ZnSe/CdSe^29^. Although our model has been applied to InAs/GaAs QD, it is a generic and can be applied to different III-V compound by considering the corresponding parameters such as band gap, potential profile, conduction and valence band offset, effective mass, and refractive index.

In our previous work of modeling of QDIPs, we have developed a model of the carrier mobilities of conical, spherical, semispherical and truncated conical QDs^30–32^. Besides, Dark current models have been developed for different QD structures in^33–35^. To enhance the optical absorption of the QD photodetectors at the wavelength of interest of IR spectroscopy, optimization of the device should be conducted to get the optimal dimensions of the QD structure and basic cell size for different QD structures.

In the presented work, Nelder–Mead simplex algorithm has been employed to maximize the optical absorption coefficient at the wavelength of interest in a multidimensional space within a defined set of physical constraints. This direct-search technique is robust and effective for handling complex non-smooth and non-differentiable problems. Therefore, an optimization study of the optical absorption coefficient of InAs/GaAs self-assembled quantum dots for IR photodetection particularly within fingerprint region is presented. The presented optimization approach is generic that can be applied for different wavelengths, different QD structures, and different QD and barrier materials. But regarding the fabrication limitations of self-assembled QDs such as surface roughness, and strain, we have focused on the most suitable shapes to model the fabricated dots, such as truncated conical, conical and semispherical shaped QDs.

The paper is organized as follows; in the section “Absorption coefficient modeling and optimization technique”, the optical absorption coefficient model for self-assembled QDs and the is presented. In addition, the applied Nelder–Mead simplex algorithm is fully described discussing the objective function, design parameters and the physical constraints. Then, the results of the optimization for the different QD structures for different wavelengths of interest have been developed and discussed in the section “Results and discussion”, where these optimization results have also been compared with previously published results. Then the sensitivity is presented in section “Sensitivities analysis of absorption coefficient of QD structures”. Finally, the conclusions have been summarized in the section “Conclusion”.

## Absorption coefficient modeling and optimization technique

**Fig. 1:**
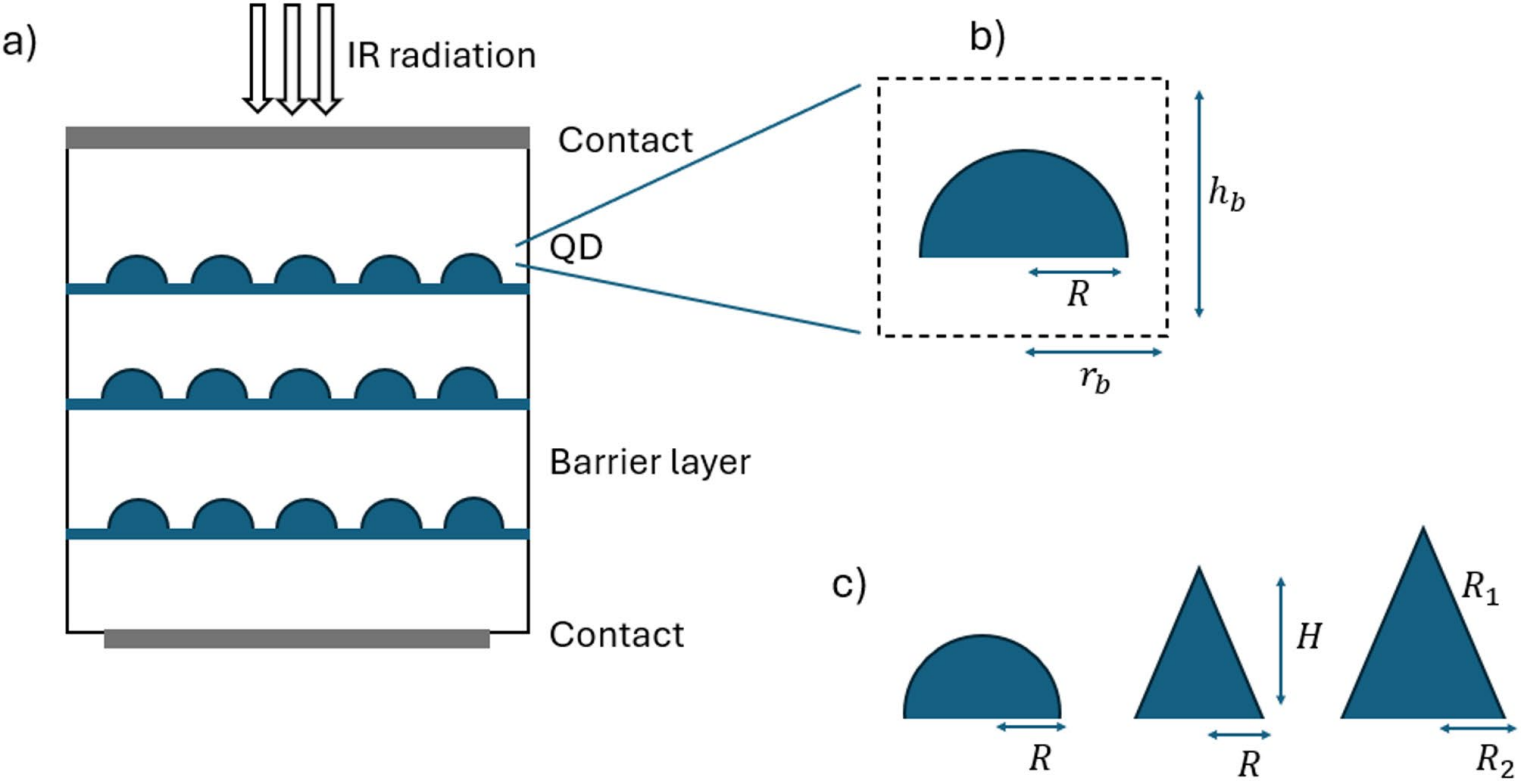
(a) Schematic of QD layers periodic structure, (b) one unit cell including the dot and the barrier, (c) different dot shapes, semispherical, conical and truncated conical.

The basic cell of the proposed QD structures is made up of multi-layers, modeled here as ten layers, of self-assembled InAs embedded in a GaAs barrier material region, where the radius and the height of the basic cell are $\:{r}_{b}$ and $\:{h}_{b}$, respectively as depicted in Fig. 1. Regarding the design parameters of the QD, they are following: (1) For semispherical QD, $\:R$ is the radius of the QD, (2) For conical QD, the radius and the height of the QD are $\:R$ and $\:H$, respectively, (3) for truncated QD, $\:{R}_{1}$ and $\:{R}_{2}$ are the radii of the top and bottom bases of the QD whereas $\:H$ is its height. For these structures, the top contact is of a transparent conducting material (TCM) like ITO, doped ZnO (Al: ZnO, In: ZnO, Ga: ZnO), F-doped SnO2 (FTO), and amorphous InGaZnO4 (IGZO), which are frequently used for optoelectronic applications^36^, or of a highly doped GaAs, which has been taken into consideration in^37,38^ with a suitable structure to permit the incidence of light. The coupling between the dots is disregarded since it is assumed that the distance between them is greater than the dimensions of the QDs. Furthermore, the implications of the wetting layer are ignored due to its thickness.

For modeling the optical absorption coefficient, the Hermitian Hamiltonian matrix is built using effective mass theory for the quantum dot and diagonalized to get the bound states and energies. Taking into consideration the axial symmetry of the presented structures: semispherical, conical and truncated conical, the Hamiltonian is as follows:

1 $$\:\widehat{H}=U\left(r,z\right)-\frac{{\hslash\:}^{2}}{2}\left(\frac{1}{r}\frac{\partial\:}{\partial\:r}\left(\frac{r}{{m}_{r}}\frac{\partial\:}{\partial\:r}\right)+\:\frac{-{n}^{2}}{{r}^{2}{m}_{r}}+\frac{\partial\:}{\partial\:z}\left(\frac{1}{{m}_{z}}\frac{\partial\:}{\partial\:z}\right)\right)\:$$

where $\:\hslash\:$ is reduced Planck’s constant, $\:n$ is the quantum number, $\:U\left(r,z\right)$ is the QD potential distribution inside the cell unit which equals the barrier potential $\:{V}_{b}$ outside the QD region and equals zero inside the QD, $\:{m}_{r}$ and $\:{m}_{z}$ are the effective mass in the radial and axial directions, respectively.

Assume that the transition occurs between the two energy levels $\:{E}_{i}$ and $\:{E}_{f}$ where incident radiation is.

$\:\underset{\_}{E}=\underset{\_}{{E}_{0}}\text{cos}\left(\underset{\_}{k}.\underset{\_}{r}-\omega\:t\right)\:$,where $\:\omega\:$ is the angular frequency and $\:k$ is the wavenumber, then the transition rate between these two states $\:{R}_{fi}$ either absorption or emission can be achieved via Fermi golden rule^39^ considering first order dipole approximation under the harmonic variation electric field by

2 $$\:{R}_{fi}=\frac{\pi\:{E}_{0}^{2}}{2\hslash\:}\left|\underset{\_}{{d}_{fi}}.\widehat{\underset{\_}{e}}\right|\delta\:\left({E}_{f}-{E}_{i}-\hslash\:\omega\:\right)$$

where $\:\widehat{e}$ is the polarization of the incident light and $\:{d}_{fi}$ is the first order dipole moment that is calculated as:

3 $$\:{d}_{fi}=q\iiint\:\underset{\_}{r}\:{\psi\:}_{f}^{*}\:{\psi\:}_{i}\:rdr\:d\varphi\:dz$$

where $\:{\psi\:}_{i}$ and $\:{\psi\:}_{f}$are the wave functions of initial and final bound state.

After multiplying by the probability of having electron in the origin state and probability of not.

having electron in the destination state (due to Pauli exclusion principle), then summing all the contributions from all the possible transitions with energy $\:\hslash\:\omega\:$ to get the effective absorption rate $\:W\left(\omega\:\right)={W}_{absorption}-{W}_{emission}$ as:

4 $$\:W\left(\omega\:\right)=\frac{\pi\:}{2\hslash\:}\:{\left|\underset{\_}{{E}_{0}}\right|}^{2}\sum\:_{i,f}\left|\underset{\_}{{d}_{fi}}.\widehat{\underset{\_}{e}}\right|\delta\:\left({E}_{f}-{E}_{i}-\hslash\:\omega\:\right)\left({F}_{i}-{F}_{f}\right)$$

where $\:F$ is the probability of having an electron in an energy $\:E$. Then, the absorption rate is multiplied by $\:{N}_{i}$ and $\:{N}_{f}$, which are the number of states having energy $\:{E}_{i}$ and $\:{E}_{f}$, respectively. Due to the degeneracy of the initial state, $\:{N}_{i}$ has a degeneracy of 2 for $\:n\ne\:0$ due to the negative values of $\:n$, while $\:{N}_{i}=1$ for the ground state $\:n=0$.

On the other hand, to get the number of states having energy between $\:{E}_{f}$ and $\:{E}_{f}+d{E}_{f}{\:(N}_{f})$ while including the effect of inhomogeneous broadening of the bound states, the density of states at a certain final state $\:{E}_{f{\prime\:}}$, which is the number of states having energy between $\:{E}_{f}$ and $\:{E}_{f}+d{E}_{f}$ per unit energy $\:{E}_{f}$, is taken as to be Gaussian function $\:D\left({E}_{f}\right)$

5 $$\:D\left({E}_{f}\right)=\frac{1}{\sqrt{2\pi\:}\sigma\:}{e}^{\frac{-{\left({E}_{f}-{E}_{f}^{{\prime\:}}\right)}^{2}}{2{\sigma\:}^{2}}}$$

6 $$\:{N}_{f}=2D\left({E}_{f}\right)d{E}_{f}=\frac{2\:d{E}_{f}}{\sqrt{2\pi\:}\sigma\:}{e}^{\frac{-{\left({E}_{f}-{E}_{f}^{{\prime\:}}\right)}^{2}}{2{\sigma\:}^{2}}}$$

Where $\:\sigma\:$is the standard deviation of the distribution and the 2 is multiplied due to the degeneracy of the excited states. Then, the bound-to-bound absorption coefficient is finally calculated according to the following Eqs^40–42^.:

7 $$\:\alpha\:\left(\omega\:\right)=\frac{2\pi\:\:\omega\:\:{n}_{dots}\:}{{n}^{{\prime\:}}{\epsilon\:}_{0}C}\:\sum\:_{i,f}{N}_{i}{N}_{f}{\left|\underset{\_}{{d}_{fi}}.\widehat{\underset{\_}{e}}\right|}^{2}\:D\left({E}_{f}\right)\left({F}_{i}-{F}_{f}\right),$$

8 $$\:\alpha\:\left(\omega\:\right)=\frac{2\pi\:\:\omega\:\:{n}_{dots}\:}{{n}^{{\prime\:}}{\epsilon\:}_{0}C}\:\sum\:_{i,f}{N}_{i}{\left|\underset{\_}{{d}_{fi}}.\widehat{\underset{\_}{e}}\right|}^{2}\:\left({F}_{i}-{F}_{f}\right){\frac{1}{\sqrt{2\pi\:}\sigma\:}\:\:e}^{\frac{-{\left({\hslash\:\omega\:-E}_{f}-{E}_{i}\right)}^{2}}{2{\sigma\:}^{2}}}$$

where $\:\alpha\:\left(\omega\:\right)$ is the frequency dependent inter-band absorption coefficient, $\:{n}_{dots}\:$ is the number of dots per unit volume, $\:n{\prime\:}$ is the refractive index of the material, $\:{\epsilon\:}_{0}$ is the space permittivity, $\:C$ is speed of light of free space.

Besides, the density of states and the states in the continuum are computed using Non-Equilibrium Greens Function (NEGF)^42^. NEGF is very useful tool in representing open boundary conditions via the non-Hermitian self-energy matrix $\:{{\Sigma\:}}^{R}$. The retarded Green’s function $\:{G}^{R}(r,{r}^{{\prime\:}},E)$ of the Hamiltonian operator $\:\widehat{H}$ is given by:

9 $$\:{G}^{R}={\left(\left(E+i\eta\:\right)I-H-{{\Sigma\:}}^{R}\right)}^{-1}\delta\:$$

where $\:\eta\:\:$adds an infinitesimal positive imaginary part to the energy, $\:\delta\:$ is the result of diagonalization of Dirac delta $\:\delta\:(\underset{\_}{r}-{\underset{\_}{r}}^{{\prime\:}})$, and the self-energy matrix $\:{{\Sigma\:}}^{R}$ is given by^40^

10 $$\:{{\Sigma\:}}^{R}={t}^{2}\left[{g}_{l}^{R}\left({p}_{i},{p}_{j}\right)\right]\left({r}_{b}{{\Delta\:}}^{2}\right)\:,\:\:t=-\frac{{\hslash\:}^{2}}{2{m}_{avg}{\varDelta\:}^{2}},$$

where $\:\varDelta\:$ is the spatial step, $\:{m}_{avg}$ is the average of the lateral effective mass of electron, $\:{g}_{l}^{R}\left({p}_{i},{p}_{j}\right)$ is a zero matrix which has non-zero elements between the points $\:{p}_{i},\:{p}_{j}$ at the boundary $\:r={r}_{b}.$ Then the density of states of the continuum is finally estimated by

11 $$\:D\left(E\right)=Tr\:\left(i\left({G}^{R}-{G}^{A}\right){\delta\:}^{-1}\right)$$

where $\:Tr$ is the trace of the matrix, $\:{G}^{A}$ is the advanced Green’s function ($\:{G}^{A}={{G}^{R}}^{*T}$), While we provide details on both bound-to-bound and bound-to-continuum absorption coefficients,, however, the optimization study in the presented manuscript focused exclusively on bound-to-bound absorption which is appropriate for IR spectroscopy applications as have been discussed earlier. For Infrared spectroscopy, optimization of the photodetector is critically needed to maximize the optical absorption coefficient at the wavelength of interest.

In this paper, Nelder–Mead simplex approach has been used for the optimization of the optical absorption coefficient of Quantum Dot infrared photodetectors. Nelder–Mead simplex algorithm is geometric based derivative free optimization method which makes it suitable for optimization of non–smooth functions, as many variants of the standard algorithm have been proposed to improve the convergence properties for small and high dimensions^43,44^. In^45^, the convergence of the matrix form of this algorithm is investigated, where an implementation of a modified Nelder-Mead algorithm for high dimensions is presented in^46^. The fact that the Nelder-Mead algorithm is derivative-free makes it suitable for optimizing computationally expensive objective functions, especially those that are “black-box” in nature^47^. For QD infrared sensing, this algorithm is used for maximizing the optical absorption coefficient ($\:\alpha\:\left(x\right)$) of InAs/GaAs Quantum dot, where $\:x$ is the design vector includes the QD dimensions and both the height and radius of the basic cell. The algorithm starts with the evaluation of the absorption coefficient $\:\alpha\:$ at $\:n+$1 points constituting simplex polygon for $\:n$-dimensional problem. The simplex is a triangle in case of *two*-*dimensional* problem and tetrahedron in case of *three-dimensional* problem. The iterative process of the algorithm continuously replaces the point with lowest value of $\:\alpha\:$ by another good point that has higher value of $\:\alpha\:$.

**Fig. 2:**
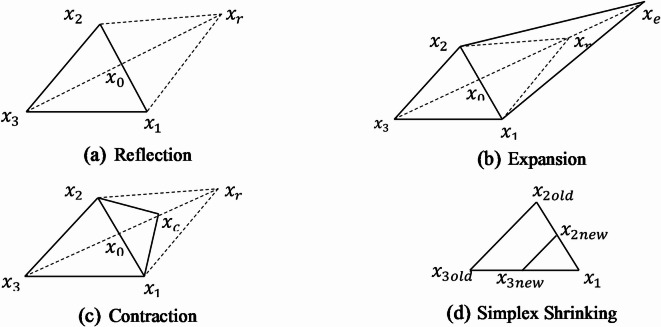
Nelder Mead Approach for Maximizing the Absorption Coefficient.

The iteration steps of the algorithm start by identifying initial suitable simplex points then ordering the simplex points $\:{x}_{1}$, $\:{x}_{2},\dots\:,{x}_{n+1}$ according to the values of $\:\alpha\:$ such that $\:{\alpha\:}_{best}=\alpha\:\left({x}_{1}\right)$ is the best value of absorption coefficient and $\:{\alpha\:}_{worst}=\alpha\:\left({x}_{n+1}\right)$ is the worst value. After that, the centroid point

12 $$\:{x}_{0}=\frac{1}{n}\sum\:_{i=1}^{n}{x}_{i},$$

for the simplex points excluding the worst point $\:{x}_{n+1}$ is calculated according to: The simplex then moves towards the optimal point by iteratively updating the simplex points through three operations: Reflection, Expansion and Contraction:

- Reflection: a new iterate point $\:{x}_{r}$ is found by reflecting the worst point around the centroid $\:{x}_{0}$:

13 $$\:{x}_{r}={x}_{0}+a\left({{x}_{0}-x}_{n+1}\right),\:a>0$$

($\:a=1$ is commonly used hence $\:{x}_{r}=2\:{x}_{0}{-x}_{n+1}$). If $\:{{\alpha\:}_{worst}<\alpha\:\left({x}_{r}\right)<\alpha\:}_{best}\:,$ the point $\:{x}_{r}$ is accepted as the new iteration point and the worst point is rejected. Figure 2 (a) illustrates the reflection process for a triangle simplex with 3 points $\:{x}_{1}$, $\:{x}_{2}$ and $\:{x}_{3}$ where the worst point $\:{x}_{3}$ is replaced by the reflected point $\:{x}_{r}$.

- Expansion: If $\:\alpha\:\left({x}_{r}\right)>{\alpha\:}_{best}$, this indicates that the direction is promising to maximize $\:\alpha\:$, hence, a new point is evaluated as:

14 $$\:{x}_{e}={x}_{0}+b({x}_{r}-{x}_{0}),\:b>1$$

($\:b=2$ is common choice). If $\:\alpha\:\left({x}_{e}\right)>\alpha\:\left({x}_{r}\right)$ then $\:{x}_{e}$ is accepted and the worst point is rejected. If $\:\alpha\:\left({x}_{e}\right)<\alpha\:\left({x}_{r}\right)$ then the expansion is not successful and $\:{x}_{r}$ is accepted. The expansion step is illustrated in Fig. 2 (b).

- Contraction: If $\:\alpha\:\left({x}_{r}\right)<\alpha\:\left({x}_{n+1}\right)$ i.e. the reflection point is even worse than the worst point then a contraction point $\:{x}_{c}$ is located inside the current simplex and is computed by:

15 $$\:{x}_{c}={(1-g)x}_{0}+g{x}_{n+1}\:,\:0<g<0.5\:.$$

If $\:\alpha\:\left({x}_{c}\right)>\alpha\:\left({x}_{n+1}\right)$, the contraction point is accepted replacing the worst point $\:{x}_{n+1}$ and shrinking the current simplex is performed, where this process is illustrated in Fig. 2 (c).

- If $\:\alpha\:\left({x}_{n+1}\right)<\alpha\:\left({x}_{r}\right)<\alpha\:\left({x}_{n}\right)$ ($\:{x}_{r}$ is better than the worst point but not better than the second worst point $\:{x}_{n}$) in this case a contraction point outside the current simplex is located as:

16 $$\:{x}_{c}={(1-b)x}_{0}+b{x}_{r}\:,\:0<b<0.5.$$

If $\:\alpha\:\left({x}_{c}\right)>\alpha\:\left({x}_{r}\right)$, the point $\:{x}_{c}$ is accepted and shrinking the current simplex is performed.

- Shrinking the current simplex: the best point $\:{x}_{1}$is left unchanged while all other points are transformed to *n* new points to shrink the current simplex to a new one. The new simplex points are given by:

17 $$\:{x}_{jnew}={x}_{1}+\delta\:({x}_{jold}-{x}_{1}),\:0<\delta\:<1$$

($\:\delta\:=0.5\:$is a typical value). The three processes reflection, expansion and contraction allow for the movement of the simplex towards the optimal solution. The shrink process of the simplex illustrated in Fig. 2 (d) allows better exploring the search space, reduces the chance of getting trapped in local optimal solution, increases the robustness of the optimization algorithm.

## Results and discussion

In this section, the optimization results of maximizing the optical absorption coefficient for certain wavenumbers of $\:600$ and $\:800$ $\:\text{cm}^{-1}$ for different QD structures are presented and discussed, where these wavenumber values have been chosen as example for IR spectroscopy applications such as some food detection (coffee^48,49^, honey^50^, cheese^51^. Besides, the applied algorithm results have been compared with published results in^40,41^, showing a considerable enhancement of the absorption coefficient at the same wavenumbers. As the proposed optimization approach and the associated model of the optical absorption coefficient is generic, then it can be applied to different structures of self-assembled QDs. But regarding the fabrication limitations of self-assembled QDs such as surface roughness, and strain, we have focused on the most suitable shapes to model the fabricated dots, such as truncated conical, conical and semispherical shaped QDs.

### Optimization of semispherical QDs

For the semispherical QD structure, the optimization technique has successfully estimated the basic cell design parameters; QD radius $\:R$, barrier radius $\:{r}_{b}$, and barrier height $\:{h}_{b}$ which achieves the maximum absorption coefficient and is superior to the published absorption coefficient at the same wavenumber.

**Fig. 3:**
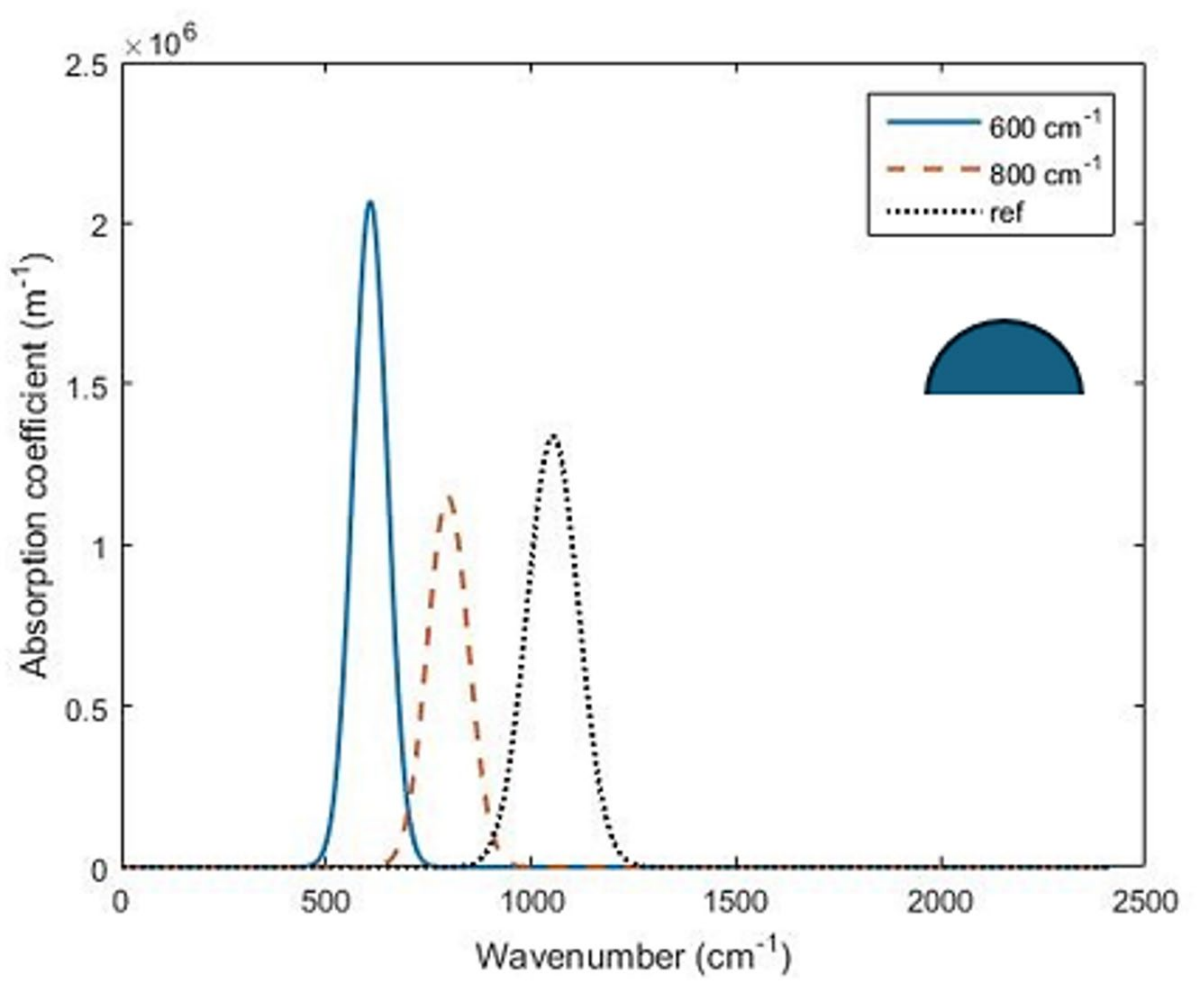
Optimized absorption coefficient for semispherical QD at different wavenumbers.

For the proposed Nelder–Mead optimization process, the initial values of design parameters considered as a starting point are taken as $\:R=10\:\text{nm}$, $\:{r}_{b}=25\:\text{nm}$ and $\:{h}_{b}=20\:\text{nm}$. The optimal values of these design parameters and the associated maximized absorption coefficient for wavenumbers of 600, 800 and 1048 cm^−1^ have been listed in Table 1. The results show a significant enhancement of the absorption coefficient compared to the initial starting solution, as a result to the application of the optimization process. Figure 3 shows the bound-to-bound absorption coefficients of the optimized cells of semispherical QDs for wavenumbers of 600, 800 and $\:1048\:\text{cm}^{-1}$. As shown in this figure, the value of the optical absorption coefficient for the optimal design increases as the wavenumber of interest decreases (increasing of the wavelength). Also, for larger wavenumber of interest, both the optimal size of the QD and the optimal barrier radius decrease, which results in a broadening of the absorption coefficient pattern via the wavenumbers. This can be explained by knowing that the bounds with the higher energy are less confined in the QD region than ground states, and it penetrates further in the barrier region^35^. Unlike spherical QDs, in the semispherical shaped QD, the symmetry between the curved and flat surfaces was broken which decreases the dipole moment $\:{d}_{fi}$ shown in Eq. (3) for asymmetric excited states. So, as the optimized dot is typically sized to maximize the bound-to-bound transition at certain energy, the absorption coefficient has less value between higher energy excited states.

To confirm the achieved enhancement of the optical absorption coefficient for the optimal design parameters, the proposed optimization technique has been applied to wavenumber of $\:1048\:\text{cm}^{-1}$ to compare the performance with a previously published work in^41^. As shown in Fig. 3; Table 1, for this wavenumber, the optimal design parameters result in improving the absorption coefficient by around 5.5 times compared to that of the QD structure in^41^.

**Table 1:** Optimized design parameters and the maximum absorption coefficient for semispherical QD Structure.

Wavenumber (cm^−1^)		$\:R\left(nm\right)$	$\:{r}_{b}\left(nm\right)$	$\:{h}_{b}\:\left(nm\right)$	Absorption coefficient $\:\alpha\:$ (m^−1^)
$\:600$		$\:11.95$	$\:12.95$	$\:13.66$	$\:2.0946\:e\:6$
$\:800$		$\:9.35$	$\:11.55$	$\:24.46$	$\:1.15\:e\:6$
$\:1048.34$	Optimal (this work)	$\:7.64$	$\:9.45$	$\:24.28$	$\:1.33\:e\:6$
	(Published in^41^)	$\:6.713$	$\:17.5$	$\:28$	$\:(2.43\:e\:5$ in^41^)

### Optimization of conical QDs

**Fig. 4:**
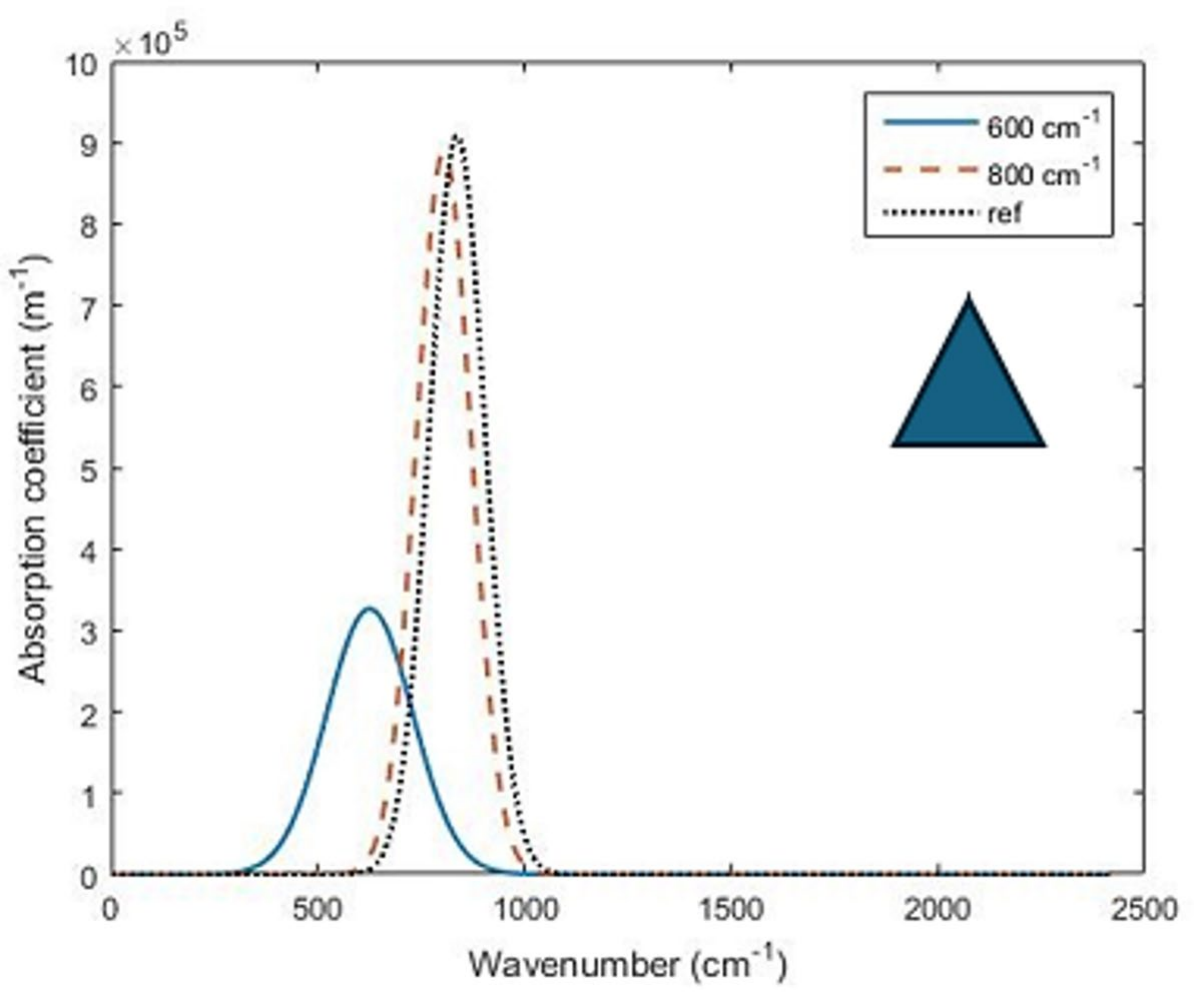
Optimized absorption coefficient for conical QD at different wavenumbers.

Regarding the conical QD shape, the parameters considered for the optimization process are the dot radius $\:R$, the dot height $\:H$, the barrier radius $\:{r}_{b}$ and the barrier height $\:{h}_{b}$, as shown in Fig. 1. The initial design parameters considered as a starting point for the Nelder–Mead optimization are taken as $\:R=10\:nm$, $\:H=28nm$, $\:{r}_{b}=17\:nm$ and $\:{h}_{b}=6\:nm$. The optimum design parameters based on Nelder–Mead simplex and the associated absorption coefficient for the wavenumber of interest at $\:600,\:800$ and $\:827\:\text{cm}^{-1}$ have been listed in Table 2. The shape and the size of the quantum dot affects the spatial distribution of the electronic states and hence affect both the wavelength of the maximum absorption coefficient and its maximum value. As shown in Fig. 4, increasing the cone height and reducing the radius, the optimum absorption spectrum is shifted to higher wavenumber and lower wavelength and this optimal absorption profile is narrowed, as increasing the cone height results in increase of the energy difference between the confined energy levels in the QD which affects the bound to bound absorption. Unlike semispherical shaped QD, conical QD has a sharp tip and wide base, creating a variant confinement potential along the axis which is stronger at the top and weak at the base. This results in overlapping between the high energy states and more dipole moment. As shown in Table 2, for a wavenumber of $\:827\:\text{cm}^{-1}$, the optimization results of the conical QD structure have been compared to the publishes results in^40,42^ showing an enhancement of the optical absorption coefficient by more than 4 times.

**Table 2:** The optimized cell parameters and maximum absorption coefficient for conical QD.

Wavenumber (cm^−1^)		$\:R\left(nm\right)$	$\:H\left(nm\right)$	$\:{r}_{b}\left(nm\right)$	$\:{h}_{b}\:\left(nm\right)$	Absorption coefficient $\:\alpha\:$ (m^−1^)
$\:600$		$\:12.02$	$\:3.01$	$\:13.01$	$\:23.94$	$\:3.412\:e\:5$
$\:800$		$\:11.64$	$\:7.76$	$\:12.64$	$\:18.84$	$\:8.86\:e\:5$
$\:827.6$	Optimal (this work)	$\:11.25$	$\:7.95$	$\:12.25$	$\:19.15$	$\:9.236\:e\:5$
	Published^40,42^)	$\:9.8$	$\:6.3$	$\:17.5$	$\:28$	$\:\left(2.15\:e\:5\right)$

### Optimization of truncated conical QDs

**Fig. 5:**
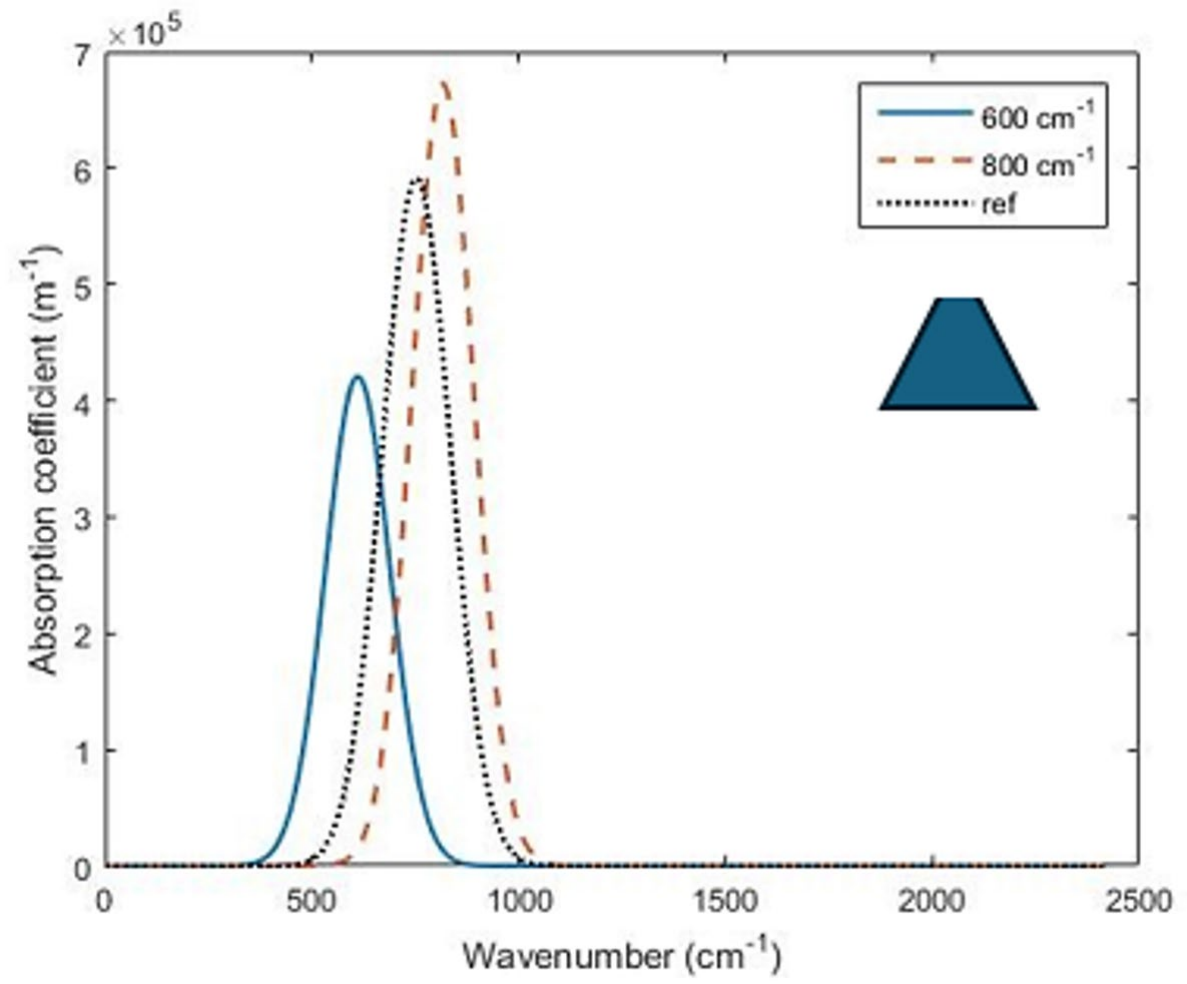
Optimized absorption coefficient for truncated conical QD at different wavenumbers.

For truncated conical QD structure, the design parameters considered for the optimization process are the QD base radius $\:{R}_{1}$, the QD top radius $\:{R}_{2}$, the QD height $\:H,$ the barrier radius $\:{r}_{b}$ and the barrier height $\:{h}_{b}$, as shown in Fig. 1. The initial values of design parameters considered as a starting point for the Nelder–Mead optimization are taken as $\:{R}_{1}=3\:nm$, $\:{R}_{2}=10\:nm$, $\:H=25\:nm$, $\:{r}_{b}=20\:nm$ and $\:{h}_{b}=10\:nm$. The optimum design parameters based on the proposed Nelder–Mead simplex and the associated absorption coefficient for the wavenumber of interest at 600, 800 and 746 $\:\text{cm}^{-1}$ have been listed in Table 3 and shown in Fig. 5. As shown in Table 3; Fig. 5, as the wavenumber of interest increases, the optimal truncated conical QD bases radii decrease, while the optimal QD height does not change effectively. Besides, the optimal barrier height and radius decrease. Comparing the results of the presented optimization of the truncated conical QD structure with the performance of a previously published work in^41^, for wavenumber of $\:746.5\:\text{cm}^{-1}$ where the optimal QD design results in an enhancement of the optical absorption coefficient by 2.75 times.

Comparing the optimization regarding the different QD structures in Tables 1, 2 and 3, it can be easily noticed that for a certain wavelength of interest, the optimized QD design of the semispherical QD structure results in a more enhancement of the optical absorption compared to the optimized QD design of either conical or truncated conical structures. This is because that the semispherical dots have a more uniform distribution, while conical and truncated conical dots have a gradient in their dimensions, leading to different confinement potentials.

**Table 3:** Values of optimized cell parameters and maximum absorption coefficient for truncated conical QD.

Wavenumber (cm^−1^)		$\:{R}_{1}\left(nm\right)$	$\:{R}_{2}\left(nm\right)$	$\:H\left(nm\right)$	$\:{r}_{b}\left(nm\right)$	$\:{h}_{b}\:\left(nm\right)$	Absorption coefficient $\:\alpha\:$ (m^−1^)
$\:600$		$\:5.27$	$\:13.25$	$\:4.25$	$\:14.25$	$\:25.07$	$\:4.716\:e\:5$
$\:800$		$\:4.1$	$\:10.68$	$\:4.88$	$\:11.75$	$\:24.74$	$\:6.78\:e\:5$
$\:827.6$	Optimal (this work)	$\:4.34$	$\:11$	$\:4.44$	$\:12$	$\:23.34$	$\:6.6\:e\:5$
	Published in^41^)	$\:4.9$	$\:9.8$	$\:4$	$\:17.5$	$\:28$	$\:2.402\:e\:5$

## Sensitivities analysis of absorption coefficient of QD structures

**Fig. 6:**
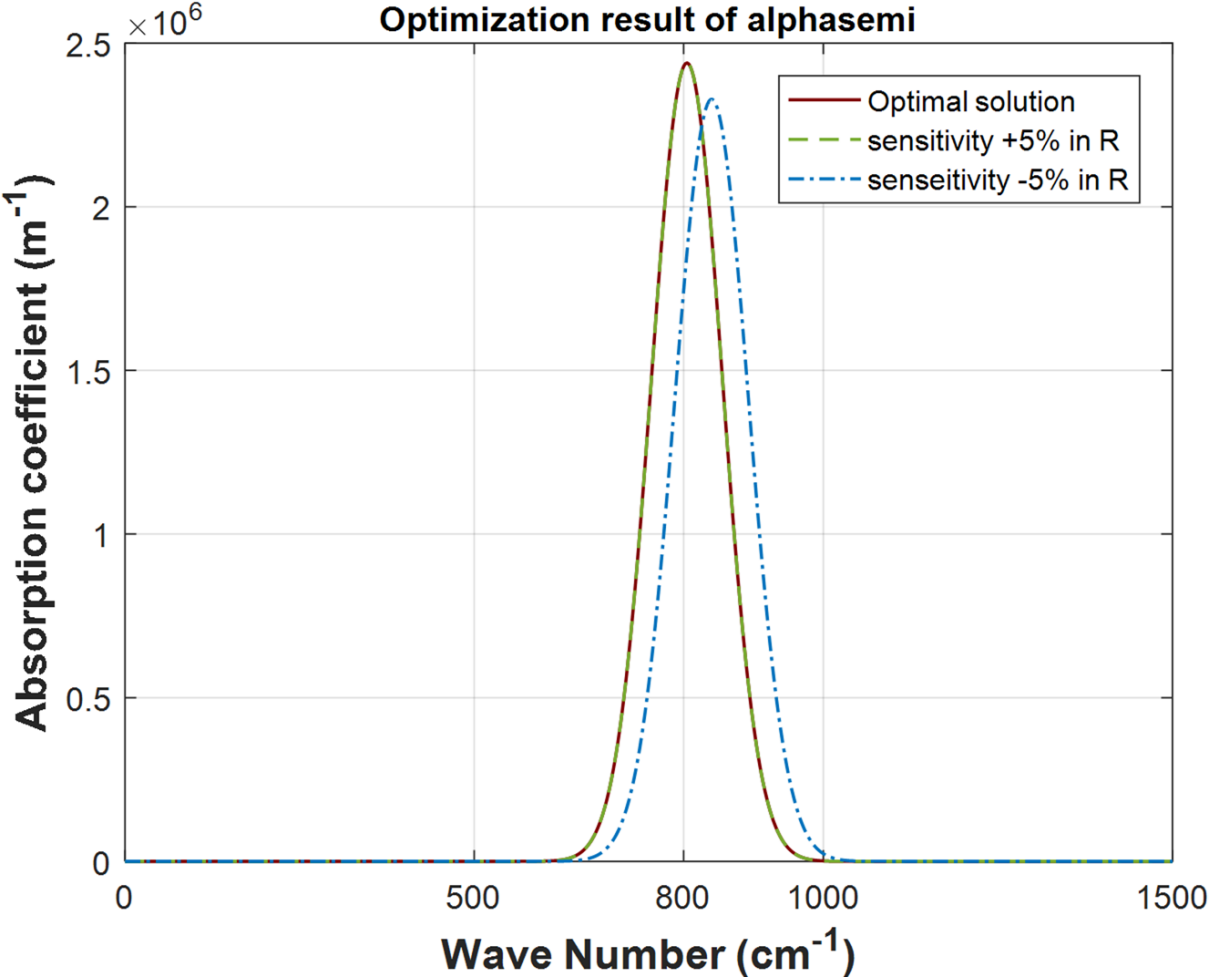
Absorption coefficient of semispherical QD with $\:\pm\:5\varvec{\%}$ sensitivity for $\:800\:\text{cm}^{-1}\:$wavenumber: a) $\:\varvec{R}$.

In this section, the sensitivity of the optical absorption coefficient of the QD photodetectors, to the main QD design parameters is studied as a guide for the allowed fabrication tolerances and to illustrate their effects on the performance of the proposed. Each parameter is perturbed with $\:\pm\:5\%$ of its optimal value at wavenumber of $\:800\:\text{cm}^{-1}$. The presented sensitivity analysis examines how variations in input variables affect the output of a model to understand the robustness of the model’s predictions and to identify critical inputs that significantly impact the results. This can guide data collection and model improvement efforts. In this study, Local sensitivity analysis is used where one input variable changes at a time while the other design parameters are kept constant to investigate its effect on the output. Regarding the sensitivity analysis of the semispherical QD structures, the effects of the $\:\pm\:\:5\%$ perturbation of the dot radius $\:R$, cell height $\:{h}_{b}$ and cell radius $\:{r}_{b}$ on the absorption coefficient are investigated in Fig. 6 (a), (b) and (c), respectively. As shown in Fig. 6, the perturbation of the dot radius has a notable influence on the peak of the absorption coefficient and results in a shifting of the wavenumber of this peak.

**Fig. 7:**
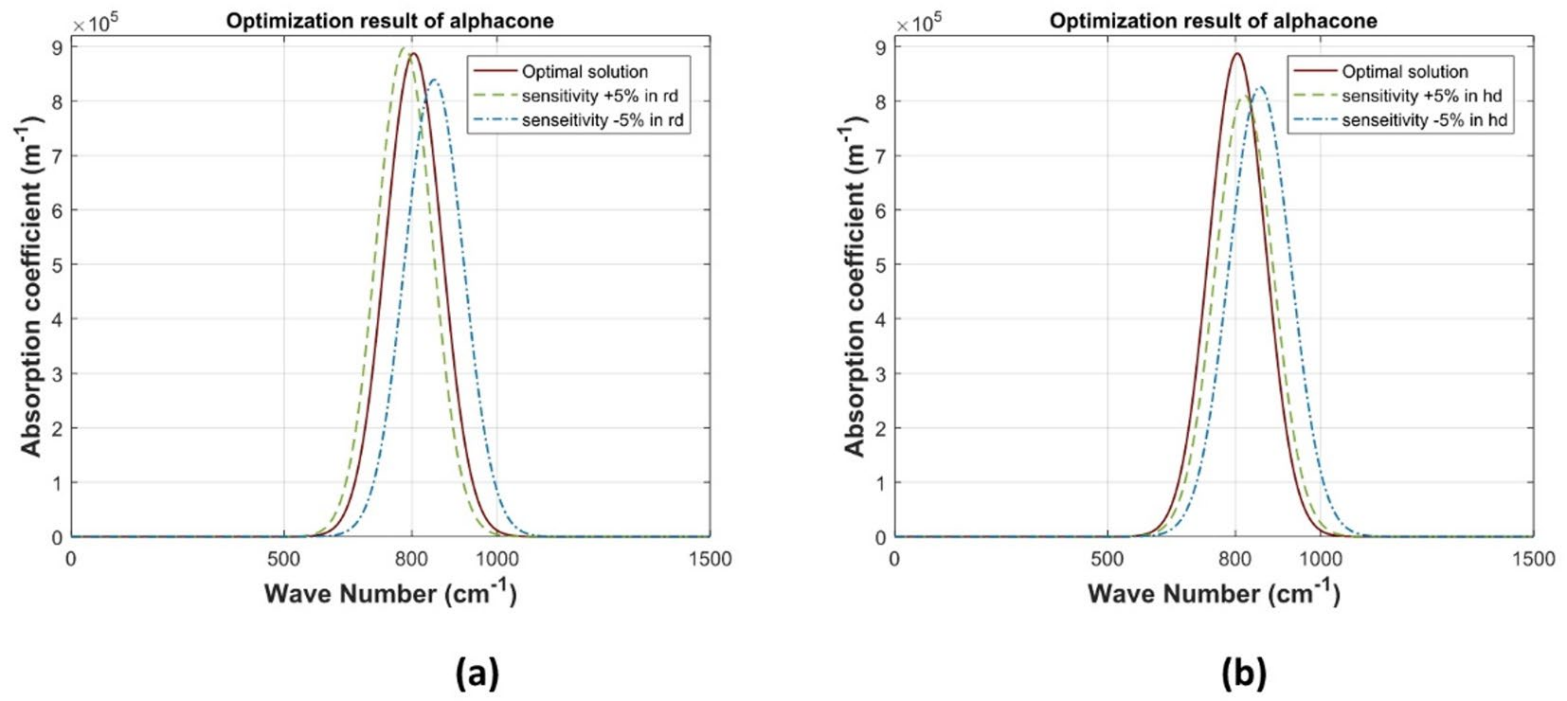
absorption coefficient of conical QD with $\:\pm\:5\varvec{\%}$ sensitivity for $\:800\:\text{cm}^{-1}\:$wavenumber: a) $\:\varvec{R}$, b) $\:\varvec{H}$.

For the Conical QD structures, the presented sensitivity analysis is based on the perturbation of the dot radius $\:R$ and the dot height $\:H$ $\:\pm\:\:5\%$ around their optimal values for maximum absorption coefficient at $\:800\:\text{cm}^{-1}$ wavenumber. The impacts of this perturbation of these design parameters over the optical absorption coefficient are illustrated in Fig. 7. The aspect ratio (height to base diameter) of conical dots can be varied more significantly than semispherical dots. This variation can lead to different absorption spectra as the electronic states are more sensitive to changes in the aspect ratio. Besides, Conical dots generally have a larger surface area relative to their volume compared to semispherical dots. This can influence the interaction with incident light and overall absorption efficiency.

**Fig. 8:**
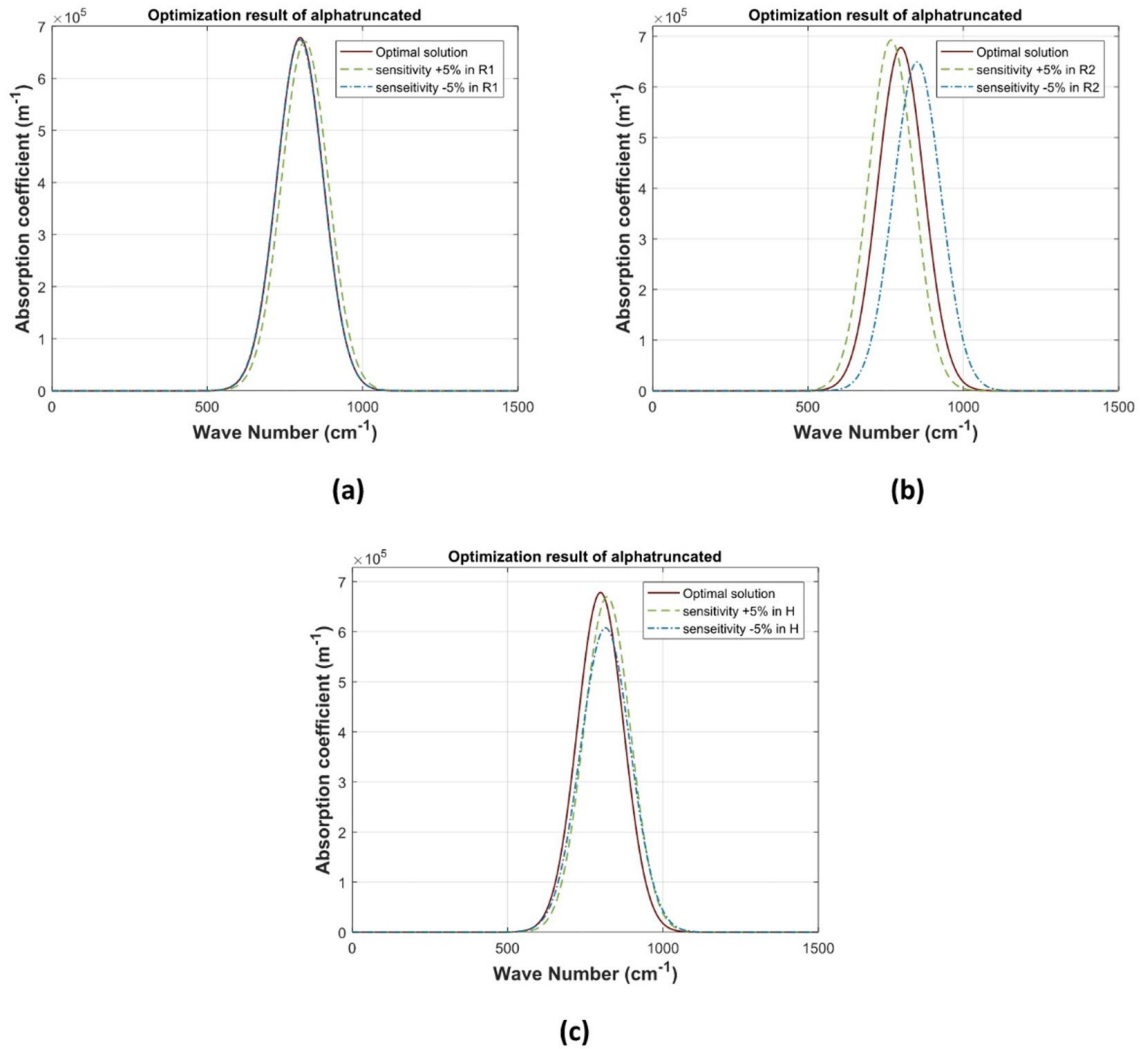
absorption coefficient of truncated conical QD a) $\:{\varvec{R}}_{1}$, b) $\:{\varvec{R}}_{2}$ c) $\:\varvec{H}$.

Different from semispherical and conical QDs, the truncated conical QD structures have larger number of design parameters including the base’s radii and the dot height in addition to the cell radius and height. The sensitivity analysis for the truncated conical QD regarding the effect of the perturbation of the QD radii and height over the optical absorption coefficient are illustrated in Fig. 8. As shown in this figure, the dot base radius has the dominant effect of the dot parameters when variation of $\:\pm\:5\%$ occurs, as the base radius controls the width of low-confinement region which correspondingly controls the high energy excited states overlapping, whereas the top radius controls the ground states (low-energy) at higher confinement and discretization. Perturbing the QD height from its value for maximum absorption coefficient, resulting in a blue shifting of the wavelength of this peak. This can be explained as decreasing the QD height results in more vertical confinement, while increasing this height leads to a change in the lateral dimensions and therefore more horizontal confinement. This can be more illustrated by Table 4 of bound states energies for the optimized and $\:\pm\:5\%$ heights (in eV), where the difference between the bound states increases for both $\:\pm\:\:$values.

As shown in Table, vertical confinement, which is represented by the $\:-5\text{\%}$, affects the ground states more than the horizontal confinement. The horizontal confinement is weaker near the base, where the ground state tends to localize, due to the larger radius, and stronger near the top. That’s why the vertical confinement, which defines the quantization along the z-axis, has the dominant effect on the lowest energy levels. On the other hand, the excited states extend into the narrower top, where horizontal confinement becomes stronger, thus affecting their energy more.

**Table 4:** Bound States energies (in eV) for the optimized and$\:\pm\:5 {\%}$ heights.

	Optimized	$\:+5\%\:in\:H$	$\:-5\%\:in\:H$
$\:n=0$	$\:0.187$	$\:0.1873$	$\:0.2009$
$\:n=1$	$\:0.2876$	$\:0.2896$	$\:0.3031$
$\:\varDelta\:E$	$\:0.1006$	$\:0.1023$	$\:0.1022$

## Conclusion

An optimization study of absorption coefficient of InAs/GaAs self-assembled quantum dots for IR spectroscopy has been developed in the presented work based on Nelder–Mead simplex optimization algorithm, where the objective function of this optimization is to maximize the associated optical absorption coefficient at certain wavenumbers of interest at $\:600$ and $\:800\:\text{cm}^{-1}$. Furthermore, the results of the applied optimization approach have been compared with previously published work showing a great enhancement in the bound-to-bound optical absorption coefficient for QD infrared sensing. Bound-to-bound absorption coefficient calculations are based on the bounded states estimation by effective mass Hamiltonian diagonalization.

Different dot shapes have been investigated including semispherical, conical and truncated conical quantum dots and the optimized absorption coefficient showed promising high values at wavelength of interest. The optimized QD design of the semispherical QD structure results in a more enhancement of the optical absorption compared to the optimized QD design of either conical or truncated conical structures, as the semispherical dots have a more uniform distribution, while conical and truncated conical dots have a gradient in their dimensions, leading to different confinement potentials. Different dot shapes, surface area and density of states influence how semispherical, conical and truncated conical quantum dots absorb light, making each suitable for different applications depending on the desired absorption characteristics.

Finally, a sensitivity analysis of the optical absorption coefficient of the QD photodetectors, to the main QD design parameters has been performed to check the allowed fabrication tolerances and to illustrate their effects on the performance of the proposed photodetectors. In this analysis, each design parameter of all the studied QD structures has been perturbed with $\:5\%$ of its optimal value. Perturbing the QD height from its value for maximum absorption coefficient, resulting in a shifted wavelength, as decreasing the height results in more vertical confinement, while increasing it leads to a change in the lateral dimensions and therefore more horizontal confinement.

## Data Availability

All data generated or analyzed during this study are included in this published article.

## Code availability

The authors declare that they have made their custom code.
